# Advancing infection profiling under data uncertainty through contagion potential

**DOI:** 10.1371/journal.pone.0329828

**Published:** 2025-08-12

**Authors:** Satyaki Roy, Preetom Biswas, Preetam Ghosh

**Affiliations:** 1 Department of Mathematical Sciences, The University of Alabama in Huntsville, Huntsville, Alabama, United States of America; 2 School of Computing and Augmented Intelligence, Arizona State University, Tempe, Arizona, United States of America; 3 Department of Computer Science, Virginia Commonwealth University, Richmond, Virginia, United States of America; Universidad Nacional de Colombia, COLOMBIA

## Abstract

During the COVID-19 pandemic, the prevalence of asymptomatic cases challenged the reliability of epidemiological statistics in policymaking. To address this, we introduced *contagion potential* (CP) as a continuous metric derived from sociodemographic and epidemiological data to quantify the infection risk posed by the asymptomatic within a region. However, CP estimation is hindered by incomplete or biased incidence data, where underreporting and testing constraints make direct estimation infeasible. To overcome this limitation, we employ a hypothesis-testing approach to infer CP from sampled data, allowing for robust estimation despite missing information. Even within the sample collected from spatial contact data, individuals possess partial knowledge of their neighborhoods, as their awareness is restricted to interactions captured by available tracking data. We introduce an adjustment factor that calibrates the sample CPs so that the sample is a reasonable estimate of the population CP. Further complicating estimation, biases in epidemiological and mobility data arise from heterogeneous reporting rates and sampling inconsistencies, which we address through *inverse probability weighting* to enhance reliability. Using a spatial model for infection spread through social mixing and an optimization framework based on the SIRS epidemic model, we analyze real infection datasets from Italy, Germany, and Austria. Our findings demonstrate that statistical methods can achieve high-confidence CP estimates while accounting for variations in sample size, confidence level, mobility models, and viral strains. By assessing the effects of bias, social mixing, and sampling frequency, we propose statistical corrections to improve CP prediction accuracy. Finally, we discuss how reliable CP estimates can inform outbreak mitigation strategies despite the inherent uncertainties in epidemiological data.

## 1 Introduction

The relentless impact of the Coronavirus disease (COVID-19), caused by the SARS-CoV-2 virus, has reverberated across the globe, claiming over 7 million lives to date [1]. Despite remarkable strides in vaccination technology, the virus’s ability to rapidly mutate raises formidable challenges to human health [2]. These transmissible and virulent strains, designated as *variants of concern* (VoCs) by the World Health Organization, continue to pose serious threats. Despite widespread implementation of social distancing and vaccination measures, the persistence of COVID-19 case numbers underscores the imperative for sustained efforts to mitigate ongoing and future outbreaks. This necessitates multifaceted approaches, incorporating pharmaceutical interventions (i.e., vaccines and drugs), as well as non-pharmaceutical measures encompassing public policies and government interventions [3–6].

In recent times, computational methods have gained prominence, leveraging the unprecedented surge in digital technology and the consequent wealth of available data [7, 8]. The collaboration among clinicians, biologists, computer scientists, and mathematicians has led to shared expertise as well as the development of models employing deep machine learning (ML), natural language processing, and epidemiology to discern the factors influencing disease spread and design mitigation strategies [9–12]. However, despite these advancements, challenges persist in accurately curbing the global spread of infectious diseases, particularly due to the asymptomatic nature of a significant fraction of newly infected cases as well as the heterogeneity in disease presentation based on sociodemographic and physiological factors [13, 14]. Research efforts, including epidemiological modeling, contact tracing applications, and incentivization of self-quarantine, aim to address these challenges but are hindered by the limited knowledge of virus shedding by carriers and associated modeling assumptions [15–19].

Accessing population-level epidemiological information is another formidable challenge due to real-world limitations like underreporting, misreporting, and testing limitations [20, 21]. Efforts to study this uncertainty have shown that the nations with high media bias, political influence, low epidemic preparedness, and overburdened testing and healthcare facilities have greater underreporting [22, 23], suggesting that the mortality numbers could be a robust indicator of contagion [20]. However, a Brazil-based study in 2020 reported widespread underreporting of COVID-19 deaths due to poor epidemiological sensitivity [24]. Despite incomplete information, others adapted ML and epidemic models to analyze pandemic trends. These include the use of natural language processing to learn symptoms, and access to testing by analyzing tweets [25], determining under-diagnosis from time-series data [26], and adaptive tracking and forecasting [27]. On the other hand, compartmental epidemic models were adapted to incorporate underreporting [28]. They show a reduced infection spread by enforcing pharmaceutical interventions [29]. The susceptible-infected-removed (SIR) compartmental model has also been adapted into a susceptible-infected (quarantined/ free) - recovered-deceased model, to account for the temporal dynamics in undetected cases [30]. Analysis of moving averaged hospitalization and death numbers in Chicago, New York City, Buenos Aires, Argentina, and Mexico City (MC) shows that the number of underreported cases could be several times the observed numbers, reducing the perceived impact of vaccinations [21]. At the same time, a hierarchical Bayesian approach was proposed to correct underreporting (false negatives) and over-reporting (false positives), by exploiting spatial correlations [31].

**Fig 1:**
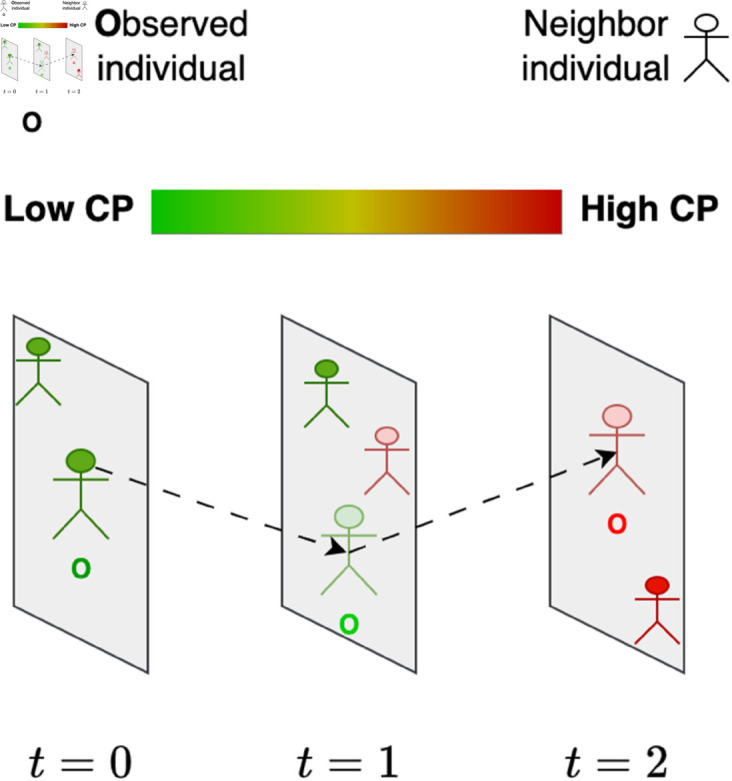
CP evolution of a person marked O (at the center of each panel). Each panel shows the person’s location at a given time. Deep green and red colors denote low and high CP values, respectively, estimated based on the CP of neighbors he interacts with.

This work is premised on the challenge of infection risk posed by asymptomatic individuals to the public. In the context of this study, *asymptomatic* refers to infected individuals who have not undergone testing and do not exhibit symptoms associated with the infection but could still act as vectors of contagion, particularly to the elderly, comorbid, or immunocompromised. We employ a *continuous* metric, termed *contagion potential* (CP), capable of quantifying the infectivity of both the symptomatic and asymptomatic as well as a population within a geographical region, based on their social contacts [32]. CP can assess an individual’s infectivity not based on their epidemiological status (tested infected or not) but in terms of the CPs of their recent contacts, modeling the diffusion of information (or infection) within a social network [33]. Specifically, a person, at the center of each panel and marked “O” in Fig 1, interacts with others over time t=1,2,3,⋯. His initial low CP (illustrated in green, close to 0) may transition to higher values (in red, close to 1) based on interactions with other individuals with high CP. Our prior analyses show that CP combines features from the network diffusion-based approaches (which use spatial contact information among individuals within a geographical region) as well as compartmental epidemic models (using population-scale epidemiology data) to estimate risks posed by the asymptomatic.

**Positioning CP within the context of current methodologies.** The existing methodologies for modeling infection transmission under uncertainty include Markov chain Monte Carlo-based Bayesian frameworks applied to partially observed spatial contact networks, which infer uncertainties in prior knowledge [34]. Stochastic agent-based models leveraging fine-grained human mobility data have been used to elucidate the spatiotemporal dynamics of contagion [35]. Additionally, approaches that jointly model viral transmission and disease progression using large-scale social network datasets have been proposed to analyze outbreaks and their associated uncertainties [36]. To address the limitations inherent in compartmental epidemic models, the Sellke construction has been employed to model the hazard of individual infection over specified periods, considering contagion risks associated with predefined epidemiological covariates [37]. This method has been utilized for survival analysis in contexts with incomplete information or lacking accurate, prior knowledge of the susceptible population [38].

As stated earlier, CP was conceived to quantify the infection risk posed by both symptomatic and asymptomatic individuals. Unlike the traditional compartmental models that categorize individuals into discrete states [39–41], CP provides a continuous measure of infectivity, capturing the nuanced dynamics of disease transmission within a population. Furthermore, the CP framework can be inferred from contact datasets, such as those obtained from mobile contact-tracing applications, as well as from population-scale, time-series incidence data. This flexibility allows for a holistic understanding of transmission patterns, especially in scenarios where data availability may be limited or heterogeneous. Overall, in contrast to survival analysis-based methods that predict individual hazards by calculating the probability of susceptibility over a predefined period, CP offers a real-time assessment of the risk posed by asymptomatic individuals. As CP is not a predictive model, it does not require exact information on infection recovery times. Instead, it generalizes the scope of diffusion in contact networks by leveraging time-series infection data, even without detailed contact information.

**Contributions.** CP was introduced in our prior works to infer the infection risk posed by symptomatic and asymptomatic individuals from multimodal epidemiology data [32, 33]. The contribution of the present work lies in extending the utility of CP beyond individual-level assessments to robust population-level inference, addressing key challenges posed by incompleteness and biases in real-world epidemiological and contact data. A fundamental challenge is the absence of complete population-level information, where underreporting and limited data availability hinder direct CP estimation. To address this, we employ t-distribution-based hypothesis testing to infer population-level CP from sampled data. However, even within the sample collected from spatial contact data, each individual has partial knowledge of their vicinity, as they can only account for neighbors with available tracking data. To mitigate this, we introduce an adjustment factor that calibrates sample-based CP estimates, ensuring they accurately reflect the true contact structure. Further compounding these issues, biases inherent in the sampling process, stemming from heterogeneous reporting rates and mobility behaviors, can distort CP estimation. To correct for these biases, we leverage *inverse probability weighting*, a statistical technique that adjusts for discrepancies in the sampling process, thereby improving the reliability of inferred CP values. By systematically addressing these challenges, our study enhances the applicability of CP in inferring sociodemographic and epidemiological patterns, reinforcing its utility for decision-making in public health.

## 2 Materials and methods

**Fig 2:**
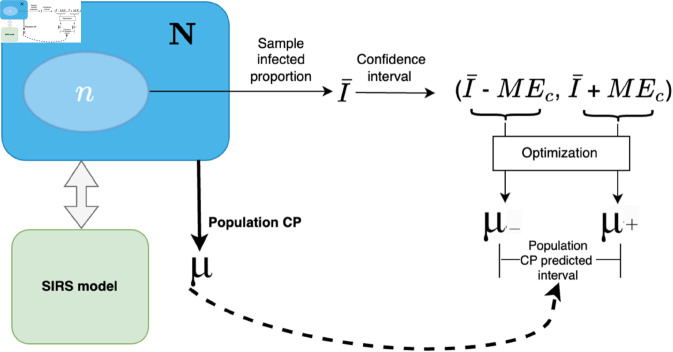
The infected fraction ($\bar{I}$) calculated on a sample of epidemiological data is used to estimate a population infected proportion confidence interval (CI) equal to a margin of error (ME) around $\bar{I}$. (Here, *ME*_*c*_ refers to the ME for a given confidence level *c*.) The upper and lower bounds for the CI are fed into the optimizer to infer a range for the estimated population CP and compared against the true CP of the population.

We consider a system of *N* individuals residing in a region, where a subset of individuals is initially infected. At each discrete time step t=1,2,…,T, the infection spreads through social contacts between susceptible and infected individuals, governed by the dynamics of the spatial or population-level Susceptible-Infected-Recovered-Susceptible (SIRS) epidemic model (refer to Sect 2.1). Concurrently, the infectivity of the population is measured in terms of CP (*μ*). A sample of the population of size *n* undergoes testing for infection (see Fig 1), and the infected proportion is denoted by $\bar{I}$. The frequency with which the predicted limits of the true CP *μ*, $\left[\mu_{-},\mu_{+}\right]$, determined by plugging $\bar{I}\pm$ margin of error (ME_*c*_) for confidence level *c* into an optimization framework (Sect 2.4), is reported (see Fig 2). The accuracy is assessed across different viral strains, human mobility models, and potential sampling biases, for an evaluation of its robustness and generalizability (see Sect 2.5 and 2.6).

### 2.1 SIRS epidemic model

**Fig 3:**
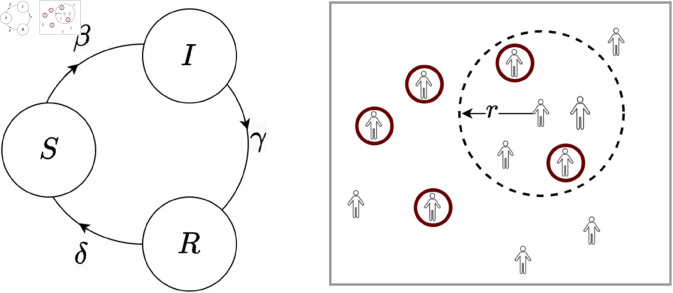
Infection spread via contact: (left) State transitions in the SIRS epidemic model; (right) individuals located in a geographical region, where the persons marked brown have location-tracking enabled. A person can track the location of individuals in his vicinity (denoted by a dotted circle of radius *r*) with tracking enabled.

We employ the Susceptible-Infected-Recovered-Susceptible (SIRS) epidemic model, as outlined by Brauer and Castillo-Chavez [39]. As enumerated in Eqs 1-3 and depicted in Fig 3 (left), a population of *N* move between three distinct classes: susceptible (S), infected (I), and recovered (R). Susceptible individuals transition to the infected class upon contact with infected individuals at a rate denoted by *β*. The infected class evolves into the recovered class at a rate *γ*, representing the recovery rate. The infection rate *β* is calculated as the product of the basic reproduction number *R*_0_ and the recovery rate *γ* [42]. The recovered individuals, however, transition back to the susceptible class with a probability of *δ*. These dynamic interactions are mathematically formalized through a system of ordinary differential equations, providing a quantitative framework for modeling the spread and recovery of infectious diseases in the population.

$$\dot{S}=-\frac{\beta SI}{N}+\delta R$$ (1)

$$\dot{I}=\frac{\beta SI}{N}-\gamma I$$ (2)

$$\dot{R}=\gamma I-\delta R$$ (3)

### 2.2 Contagion potential

As discussed in Section 1 and illustrated in Fig 1, contagion potential (CP) measures the infection risk posed by a single or a group of asymptomatic individuals located in a geographic region at a given time.

#### 2.2.1 Individual contagion potential

Contagion potential (CP) of an individual *u* (with a set of neighbor individuals v∈N(u)) at time *t* + 1 is given by:

$$\mu_{t+1}(u)=\zeta \times \mu_{t}(u)+\eta \sum_{v\in N(u)}\mu_{t}(v)$$ (4)

In the above equation, the parameter ζ is a measure of the temporal decay in CP over time, while η denotes the individual’s susceptibility to contagion as a result of social contact. There is a hard boundary on the CP values to be within the range [0,1] by performing the following operation after each update: μ=max(0,min(1,μ)).

#### 2.2.2 Zonal contagion potential

The contagion potential (CP) of a region at time *t* is defined as the *mean* CP of all individuals present in that region at time *t*. Specifically, we derive zonal CP from both human contact data and bulk epidemiological data. Incorporating both modalities allows for a comprehensive assessment of infection risk based on the collective presence of individuals and the availability of data on localized interactions and mobility patterns.

Estimating CP in both spatial and bulk settings presents unique challenges. *First*, in the spatial model, we address the uncertainty arising from the fact that individuals in the dataset may only have partial knowledge of their local neighborhood, i.e., they are only aware of contacts whose location tracking is enabled via wearable or mobile devices (see Sect 2.3). *Second*, in the bulk model, zonal CP estimation is necessary due to the lack of direct contact information between individuals. In this case, aggregated epidemiological statistics and inferred mobility patterns must be leveraged to estimate contagion potential accurately across different zones (see Sect 2.4).

### 2.3 Prediction of CP from spatial contact data

We consider a scenario in which *N* individuals are situated (and can move) within a region depicted in Fig 3 (right). An individual can locate neighbors with location-tracking enabled in their region of interaction, demarcated by a circle of radius *r*.

#### 2.3.1 Expected number of contacts

We consider a population density (measuring the ratio of the number of individuals to the area of the region) of *ρ*, which influences the average number of contacts for an individual at any given time. As shown in Fig 3 (right), and under the homogeneous mixing model [43], the expected number of individuals within the proximity of a person, defined by a circular interaction region of radius *r*, is given by $E=(\pi \times r^{2})\times \;\rho$.

#### 2.3.2 New infections based on binary and continuous infectivity

We define a binary infectivity status for individuals, denoted as 1 for tested infected and 0 for non-infected persons, resulting in a mean infectivity of $i=\frac{I}{N}$. In the second scenario, infectivity, measured by a person’s contagion potential (CP), is a continuous value within [0,1], and the population’s mean CP is represented by *μ*. The estimation of the *number* of new infected individuals at a given time (while dropping the time variable *t* in the interest of simplicity) is: ν=β×μ×S. In the real world, we do not have complete information on contacts. We assume that there is a subset of *n* individuals (in the population of *N*) whose location can be tracked with a mean sample CP $\bar{x}$ calculated from spatial contact and a sample standard deviation *s*.

#### 2.3.3 Adjustment term for incomplete contact information

Fig 3 (right) shows individuals located in a region, where the persons marked brown have location-tracking enabled. A person can track the location of individuals in their region of interaction (dotted circle of radius *r*) with tracking enabled. Since only a subset of *n* individuals can be tracked, the resultant social contact data is incomplete, making the sample CP $\bar{x}$ likely to be a poor estimate of the population statistic *μ*.

To address the challenge of untracked neighbors, we introduce an adjustment term ι capturing the discrepancy between the CP estimated from incomplete information and the true sample estimates. Parameter ι depends on sociodemographic factors, such as population density, contact rates, etc., and is estimated as the mean difference between the CP estimated from incomplete information and sample CP across tracked individuals, i.e., $\iota =\frac{\sum_{u\in 𝐧}({\bar{x}}_{u}-{\bar{x}}_{u}^{e})}{n}$, where ${\bar{x}}_{u}$ and ${\bar{x}}_{u}^{e}$ are the sample CP estimated from complete and incomplete information, respectively. Overall, after learning ι, the adjusted sample CP is calculated by adding ι to the sample CP inferred from partially observed contact data. The confidence interval for the zonal mean CP (*μ*) is calculated on the adjusted CP.

### 2.4 Prediction from bulk population data

As discussed in Sect 2.2, a zone’s mean contagion potential (CP) is the mean CP of individuals located in that zone, estimating the infected proportion of that region based on time-series incidence data. This section utilizes an optimization framework to determine CP without human contact information, using daily infection and recovery.

#### 2.4.1 CP estimation as an optimization problem

The optimization framework utilizes the population-level data on *daily* counts of infected ($I_{t}^{d}$) and recovered ($R_{t}^{d}$) individuals to estimate the mean contagion potential ($\mu_{t}$) at time *t* for each zone. (Note that $I_{t}^{d}$ represents the daily reported infections from epidemiological data and differs from the current infected at time *t*(*I*_*t*_) such that $S_{t}+I_{t}+R_{t}=N$.) The objective function (Expression 5) minimizes the error term *ε*, ensuring that the sum of susceptible $\varsigma_{t}=\frac{S_{t}}{N}$, infected $i_{t}=\frac{I_{t}}{N}$, and recovered $r_{t}=\frac{R_{t}}{N}$ proportions at time *t* is close to 1, consistent with the SIRS model structure (see Constraint 6). This constraint considers the current susceptible to be $\varsigma_{t}=\frac{\nu_{t}}{\beta \times \mu_{t}\times N}$ since the number of *new* infected $\nu_{t}=\beta \times \mu \times S_{t}$ (refer to Sect 2.3.2), while Constraint 7 considers realistic bounds $\left[\beta^{-},\beta^{+}\right]$ for the disease transmission rate *β*.

$$\min_{\beta ,\nu_{t},r_{t}}\epsilon \;$$ (5)

$$\text{s.t.}\;\frac{\nu_{t}}{\mu_{t}\times \beta \times N}+i_{t}+r_{t}+\epsilon =1\;$$ (6)

$$\beta^{-}\leq \beta \leq \beta^{+}$$ (7)

The following optimization problem helps infer the recovery rate parameter *γ* (if unknown). The number of current infected individuals at time *t* (*I*_*t*_) is the total difference between the daily infected and recovered individuals till time *t*:

$$I_{t}=\sum_{\tau =1}^{t}I_{\tau }^{d}-\sum_{\tau =1}^{t}R_{\tau }^{d},$$ (8)

The daily recovered count at time *t* ($R_{t}^{d}$) is the fraction of the current infected population, i.e., $R_{t}^{d}\approx \gamma \cdot I_{t}$, where *γ* is estimated by minimizing the squared deviations between the observed and estimated daily recovered numbers (Expression 9 and Constraint 10).

$$\min_{\gamma }\sum_{t}{\left(R_{t}^{d}-\gamma \cdot I_{t}\right)}^{2}$$ (9)

s.t. 0≤γ≤0.05 (10)

It is worth noting that this optimization is conducted separately for each zone to account for variations in incidence data availability at a localized level.

#### 2.4.2 Incomplete epidemiological information

In most practical scenarios, the population standard deviation *σ* is unknown. Consequently, confidence intervals for CP estimation are computed using the t-distribution:

$$CI(\alpha )=\left(\bar{I}-t_{\frac{\alpha }{2},n-1}\times \frac{s}{\sqrt{n}},\bar{I}+t_{\frac{\alpha }{2},n-1}\times \frac{s}{\sqrt{n}}\right),$$ (11)

In the above equation, $t_{\frac{\alpha }{2},n-1}$ is the t–score for the given confidence level *α*, with *n*−1 degrees of freedom. The parameter α=1−c, where *c* is the confidence percentage expressed as a fraction. During experiments, we represent the population of each zone as a binary vector, where each entry corresponds to an individual’s state (infected or not). To estimate the sample proportion $\bar{I}$, we randomly sample a subset from this vector, computing the fraction of infected individuals in the sample. This approach ensures that the confidence intervals reflect uncertainty in observed prevalence rates.

The population-level analysis considers the epidemiological information of a sample of *n* individuals in the population of *N*. It collects the infected fraction of the sample $\bar{I}$ to calculate the confidence interval CI(α) of the population infection proportion *I* based on the t-distribution (see Sect 2.4). For a given confidence level *c*, the extremes of the CI equal to a margin of error (*ME*_*c*_) around $\bar{I}$, i.e., $\bar{I}-ME_{c}$, $\bar{I}+ME_{c}$, are then plugged into the optimization formulation (refer to Sect 2.4.1) separately to calculate the range of values for the true population CP (denoted by $\mu_{-}and\;\mu_{+}$ illustrated in Fig 2). Finally, the accuracy of the model is measured in terms of the fraction of times the estimated interval $(\mu_{-}$ and $\mu_{+}$) includes the ground truth of the true population CP *μ*.

### 2.5 Human mobility models

In addition to the random movement of individuals from one zone (represented by a spatial grid) to another, we consider the following two human mobility models during the spatial analysis.

#### 2.5.1 Least action trip planning

This mobility model operates on the premise that humans often prioritize distance as a critical criterion in determining their next destination, referred to as a *waypoint* [44]. In essence, the likelihood of an individual selecting a specific waypoint is directly proportional to its proximity to their current location. Given a current waypoint *z*, the probability of choosing waypoint $w_{i}\in 𝐖$ is defined as:

$$p_{w_{i}}=\frac{d(z,w_{i}{)}^{-a}}{\sum_{w_{j}\in 𝐖}d(z,w_{j}{)}^{-a}}$$ (12)

Here, *d*(*z*,*w*_*i*_) represents the Euclidean distance between *z* and *w*_*i*_, and *a* is a positive constant, the weighing factor, characterizing the preference for waypoints. When *a* = 0, all waypoints have an equal likelihood of being visited, while increasing *a* assigns higher probabilities to closer waypoints. We adopt *a* = 1.2 based on the observation that LATP yields mobility traces closely matching real GPS traces within a defined range [45].

#### 2.5.2 Effect of superspreader events and variants

Superspreader events are characterized by large gatherings where individuals are exposed to the virus near potentially infected individuals. To model these events, we employ a class of human mobility models known as the Human Cell Mobility Model (HCMM) [45, 46]. According to this model, individuals, being part of social communities, are inclined to visit locations inhabited by members of their social group. The affinity of person *j* to visit location (or grid) *z* is determined by the following calculation:

$$A(z,j)=\frac{\sum_{k:k\in H_{z}}M_{j,k}}{\left|\{k:k\in H_{z}\}\right|}$$ (13)

Here, $k\in H_{z}$ represents a list of individuals, whose homes are located in grid *z*. The term $M_{j,k}\in [0,1]$ quantifies the measure of social association of person *j* towards person*k*. Two points deserve attention:

1. Consistent with social network-based models like HCMM, human mobility decisions are shaped by interactions within one’s social group. Superspreader events, characterized by large gatherings, create situations where the unvaccinated or immunocompromised may be exposed to the virus.
2. The diagonal elements of *M* conform to *M*_*j*,*j*_ = 1, and $M_{j,k}\neq M_{k,j}$ holds true if i≠j.

Another determinant of the virus’s transmissibility and virulence is its strain. We represent the infectivity of strains by integrating their basic reproduction number *R*_0_ into the rate parameter *β*, formulated as $\beta =R_{0}\times \gamma$ [42]. Recall that *γ* represents the transition rate from the infected to the recovered states.

### 2.6 Inverse probability weighting

It is a statistical method used in observational studies to estimate causal effects in the presence of confounding and selection bias [47]. It involves assigning weights to observations based on the inverse of their estimated probability of receiving the treatment or exposure. In our context, a selection bias exists when a person located at zone *u* is likely to be sampled with a likelihood score $\omega_{u}$, the concept of inverse probability weighting (IPW) comes into play. Instead of computing the simple mean of CPs from *n* sampled individuals, IPW entails calculating the mean as the inverse-weighted sum of their CPs. Given the current location and CP of individual *i*, *z*_*i*_ and $\omega_{z_{i}}$, this mean can be expressed as follows:

$$m=\frac{1}{\sum_{i=1}^{n}\omega_{z_{i}}^{-1}}\times \sum_{i}\omega_{z_{i}}^{-1}\times \mu_{i}$$ (14)

### 2.7 Datasets

We consider population-level epidemiological data of the daily COVID cases in Germany, Italy, and Austria between January 1, 2022, and June 20, 2022, obtained from Our World in Data [48]. This dataset includes cumulative positive cases, cumulative deceased cases, cumulative recovered cases, current positive cases, hospitalization figures, intensive care data, etc., categorized by date and region within each country. The dataset (of population-level epidemiological statistics of (a) Italy 1 Jan 2022 - 13 Nov 2022, (b) Germany between 1 Jan 2022 - 30 June 2022, and (c) Austria between 1 Jan 2022 - 20 June 2022) and associated Python scripts are available on https://github.com/satunr/COVID-19/blob/master/Uncertainty_CP/. We maintain a sample size above 30 to ensure that statistical inferences drawn from the data remain valid and a reliable representative of the underlying population characteristics. The confidence intervals of 90 %, 95 %, and 99 % reported in the results section (Sect 3) correspond to confidence levels of c=(1−α)=0.90,0.95,0.99, respectively, in Eq 11. The default parameter values are in Table 1. The infectivity η is measured as the ratio between the transmission rate *β* and contact rate *C* since the transmission rate β=η×C [43].

**Table 1:** Configurable experiment parameters and their default values.

Parameter	Value
Infection (I) to recovered (R) rate (*γ*) [49]	0.05
Recovered (R) to susceptible (S) rate (*δ*) [50]	0.025
CP parameter (infectivity decay rate ζ) [51]	0.32
Reproduction no. (*R*_0_) for strains Alpha [52, 53], Delta [54], and Omicron [55]	2.0,3.2,9.5
Contact radius (*r*) [56–58]	1.82 meters (or 6 feet)

## 3 Results

### 3.1 Spatial analysis

The first analysis aims to study whether we can infer an estimate of the mean population contagion potential (*μ*) from sample statistics, with varying confidence, and for different human mobility models and virus strains. We experiment over 60 days on a population of 5000 individuals, 5% of whom are initialized as infected and the remaining is susceptible. The urban space of area 2000×2000 square meters is divided into 16 square grids of equal area. Individuals migrate from one grid to another based on transition matrices following prespecified mobility models, namely LATP, HCMM, or random (refer to Sect 2.5).

#### 3.1.1 Complete contact information

We predict the confidence interval (CI) of the CP of the population based on sample CP and a prespecified confidence level. As discussed in Sect 2.4, in the real world, the standard deviation of population CP is likely to be unknown, necessitating the use of the t-distribution to determine the CI for the population CP.

**Fig 4:**
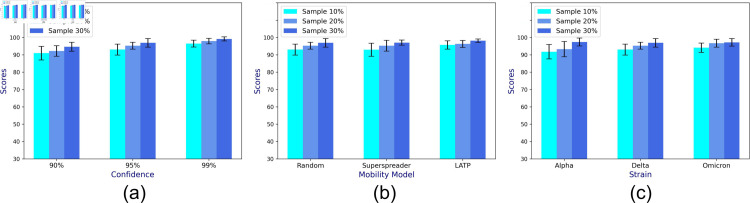
The mean prediction accuracy scores of CP (in percentage) based on complete contact information of the collected sample for varying (a) confidence intervals, (b) mobility models, and (c) viral strains.

Out of 20 runs, we measure accuracy by recording the fraction of times the sample CP’s confidence interval (CI) incorporates the population CP *μ*. To demonstrate the generalizability of the approach, we consider the following three parameters: *CI levels* varying between 90% - 100%, 3 *mobility models* (LATP, superspreader, and random), and three *virus strains* (alpha, delta, and omicron) that differ in reproduction numbers (refer to Table 1). While one parameter is varied, others assume their default values (95% CI level, random mobility, and Delta variant). Figs 4a, 4b, and 4c show that for varying CI levels, mobility, and strains, the prediction accuracy of *μ* increases with the sample size of 10% - 30% of the total population.

#### 3.1.2 Incomplete contact information

**Fig 5:**
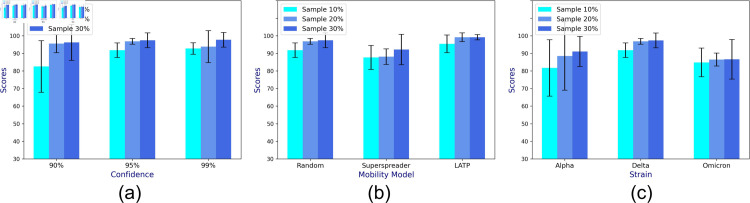
The mean prediction accuracy scores of CP (in percentage) based on incomplete contact information of the collected sample for varying (a) confidence interval, (b) mobility model, and (c) viral strain.

Since location tracking is enabled for a subset of individuals in the real world, each individual can only locate the neighbor whose location tracking is enabled. The CP estimated from incomplete contact information is unlikely to reflect the true sample as well as the population CP dynamics. We account for this dearth of information by incorporating an adjustment term to the incomplete CP estimate (as highlighted in Sect 2.3.3), before calculating the confidence interval on the adjusted CP and recording the prediction accuracy. Once again, we record the accuracy in predicted CP over 20 runs for varying confidence interval levels, mobility model, and viral strain. Unsurprisingly, Figs 5a, 5b, and 5c show that the accuracy ranges between 90–100%, exhibiting high variability (ranging from 80*to*100%) for under varying viral strains.

### 3.2 Bulk analysis

As illustrated in Sect 2.4.2, the epidemiological data is often incomplete, and the CP estimation is based on a sample of the total population. We leverage synthetic data generated using the SIRS epidemic model (see Sect 2.1) as well as the real epidemiological data from Italy, Germany, and Austria to validate whether we can define an accurate interval of the true population CP with a high degree of confidence.

**Fig 6:**
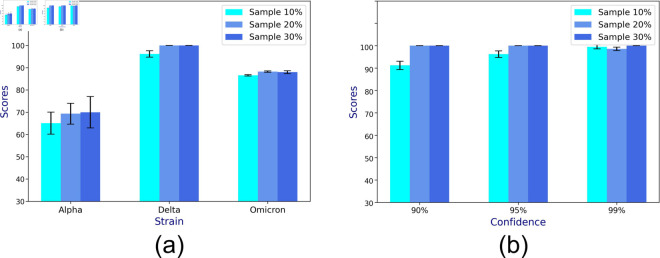
The mean prediction accuracy scores in CP (in percentage) from bulk epidemiological data across 20 runs: synthetic data generated using the SIRS model for varying (a) strains, and (b) confidence levels.

Figs 6a and 6b show the prediction accuracy of synthetic data for different strains (i.e., Alpha, Delta, and Omicron) and confidence levels (i.e., 90%, 95%, 99%) across 20 runs, while varying the sample sizes to 10%, 20%, 30% of the population. For both scenarios, there is notable variability in the accuracy of the Alpha strain. Overall, the mean accuracy ranges between 70%*to*100% and increases with sample size.

**Fig 7:**
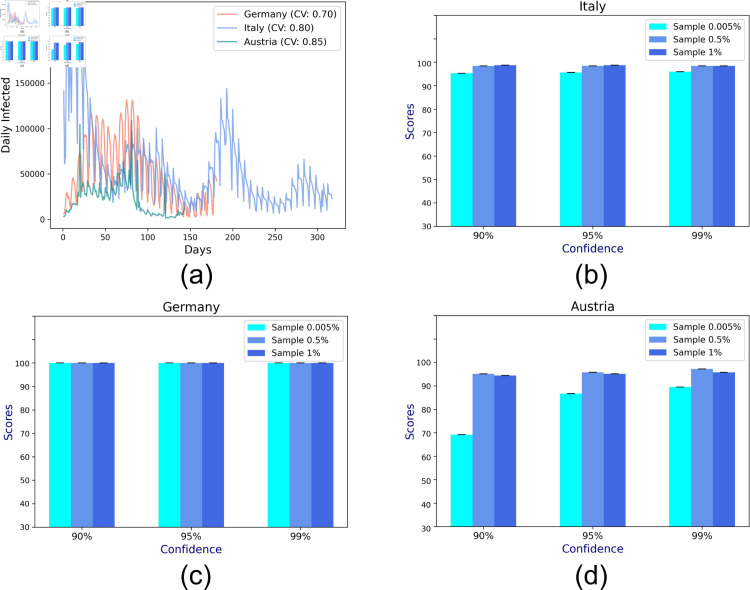
The mean prediction accuracy scores for the bulk epidemiological data across 20 runs: (a) daily infection numbers for the three countries from Jan 1st, 2022 along with their coefficient of variation; mean accuracy scores of CP (in percentage) from (b) Italy, (c) Germany, and (d) Austria.

Fig 7a shows the daily infection numbers for the three countries, namely, Italy, Germany, and Austria. For the incidence data of each country, we report the *coefficient of variation* (CV), which is the ratio of the standard deviation to the mean, providing a standardized measure of variability in daily infections, allowing for meaningful comparisons across different mean infection rates. The error bars (in Figs 7b, 7c, 7d) show that the variability in CP interval prediction accuracy for varying confidence levels in Italy, Germany, and Austria, respectively, are low. Even small fractions (0.005%, 0.5%, 1%) of the countries’ populations form a large sample size, bringing down the variability in CI. The predictive accuracy is high (~90−100%) under almost all scenarios. Austria, due to its higher variability in infection numbers (as indicated by its CV), suffers a poorer accuracy for 90% confidence level, exhibiting a high accuracy for 95% and 99% CI.

### 3.3 Effect of sampling bias

**Fig 8:**
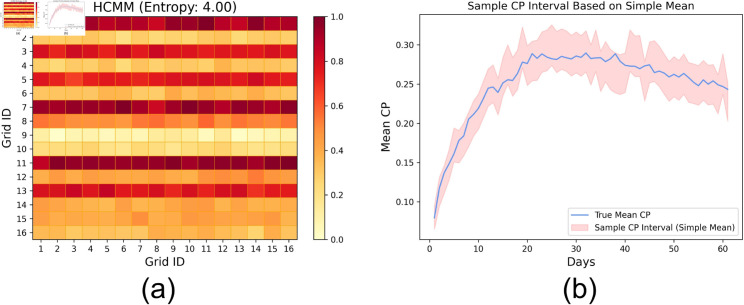
Effect of sampling bias for random social mixing: (a) Mean transition probability across 60 runs based on the HCMM mobility model; and (b) Confidence interval of estimated mean CP based on sample mean CP.

In the experiments so far, we considered random samples free from bias, making the sample a good representation of the underlying population. We now investigate the effect of such a bias on the overall CP prediction accuracy by assigning a selection probability of 0.3to two zones and 0.0286 to the remaining ones. In the first analysis, the population of 5000 individuals follows the HCMM mobility model (refer to Sect 2.5.2) to move around 16 grids. The heatmap in Fig 8a represents the mean probability of transitioning from one grid *i* to another *j*(*p*_*i*,*j*_) across 60 days. We also report the mean of row-wise entropy $E_{HCMM}=\frac{1}{16}\times \sum_{i=1}^{16}\sum_{j=1}^{16}p_{i,j}\;\times \;\log\frac{1}{p_{i,j}}$ measuring the extent of randomness or social mixing among the individuals. Fig 8b shows that despite the sampling bias, the CP prediction accuracy across 60 runs is high (∼98%).

**Fig 9:**
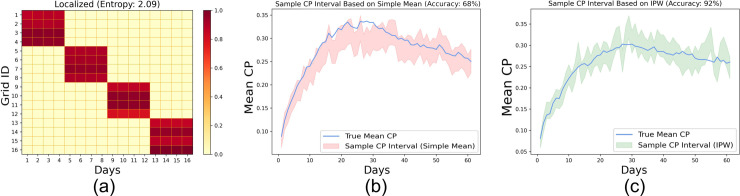
Effect of sampling bias for localized mobility: (a) Mean transition probability across 60 runs based on the localized mobility model; Confidence interval of estimated mean CP based on sample mean CP for the sample mean CP estimated by (b) simple averaging of individual CPs and (c) inverse probability weighted averaging.

To understand whether the social contact or mixing governed by the choice of mobility model plays any part in the accuracy in scenarios of sampling bias, we consider a customized, localized mobility model, where individuals are confined to move within prespecified adjacent blocks with 99% probability and are free to travel anywhere with a 1% probability (see Fig 9a). Fig 9b depicts that in the case of localized mobility, the prediction accuracy drops to 68% due to the absence of adequate social mixing. The phenomenon is further highlighted by the lower entropy (or randomness in localized mobility) *E*_*loc*_ = 2.09 than that of HCMM *E*_*HCMM*_ = 4.00. Overall, evidence suggests that the extent of social mixing can result in poor CP estimates when calculated on biased samples. Finally, we investigate whether adjusting the sample CP through inverse probability weighting (IPW), as discussed in Sect 2.6, where the sample CP of a zone is weighted by a factor equal to the inverse of its sampling probability. Fig 9c depicts that the application of IPW offsets the effect of the sampling bias, improving the CP prediction accuracy (∼92%) over a simple average-based CP estimation.

### 3.4 Variability in sample collection

**Fig 10:**
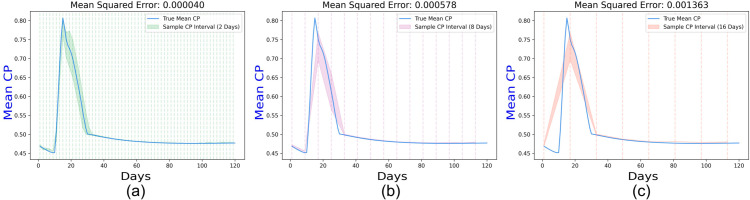
A temporal measure of the true CP and confidence interval of population CP for a frequency of sample collection of (a) 2 days, (b) 8 days, and (c) 16 days, depicted in red vertical dotted lines. Each subfigure includes a mean squared error between the true CP and sample mean CP over 120 days, showing that frequent sample collection improves prediction accuracy.

We simulate an outbreak in a spatial setting involving 100,000 individuals in an area of 2500×2500 square meters. The SIRS epidemic model is initialized with a 5% infected proportion and a fixed basic reproduction number *R*_0_ = 3.2 but a varying contact rate from 0.25 to 0.75 between days 10 and 25 to simulate an outbreak, peaking when total infection counts reached their maximum (see Fig 10a). We sample 20% of the population every I = 2, 8, 16 days and predict the 95% confidence interval (*CI*) for the mean population CP *μ*. Figs 10a, 10b, and 10c show the true and predicted CI of *μ* along with the frequency of sample collection of (a) 2 days, (b) 8 days, and (c) 16 days, depicted in vertical dotted lines. To ensure adequate readings for the 16 days, we consider an extended simulation period of 120 days. To account for the reduction in the number of readings with lower sampling frequency, we have used the Python SciPy interpolation package [59] to impute intermediate values, before reporting the mean squared errors between sample and population CPs. Frequent data collection (∼2 days) is marginally more sensitive to the evolving infection trends, underscoring the significance of sampling frequency in tracking contagion trends over time. The increase in the mean squared error with reduced sampling frequency emphasizes the importance of frequent data collection to avoid missing infection peaks and accurately estimate CP.

## 4 Discussions

This paper shows the applicability of CP for infection profiling under real-world constraints in data availability. We shall explore the following extensions. (A) *Dynamic modeling of strain-specific CP* to study the transmission characteristics to unravel how variations in viral properties influence CP over time and develop early warning systems based on confidence level estimates; (B) *disease transmission due to fine-grained interactions within closed spaces*, such as hospitals, building lobbies, and supermarkets, are characterized by fine-grained interactions among individuals. CP accounts for the varying degrees and duration of human interaction, allowing a precise assessment of transmission risk and reflecting the reality that not all interactions contribute equally to the spread of infection. Consequently, CP can inform targeted interventions and policies tailored to specific environments, improving the management of risks in public spaces where the frequency and nature of contact are diverse and complex; (C) *generalizability of the CP framework* to incorporate features from existing dynamic survival analysis based models to predict an individual’s hazard from exposure; and (D) *integration of behavioral factors* such as the public’s adherence to health measures, vaccine uptake, and societal mobility patterns, etc. Incorporating these considerations into the CP model enables a holistic understanding of spread dynamics at the population level and a finer granularity at the individual level. Such an analysis will not only enhance the model’s predictive capabilities but also provide insights for public health interventions tailored to human behaviors for socially-informed disease management; and (E) *long-term impact assessment*, where looking beyond immediate trends during outbreaks, understanding the lingering effects on communities and healthcare systems is critical for health planning. This perspective will consider factors like the buildup of immunity and the success of vaccination campaigns during seasonal outbreaks with varying spread dynamics.

## 5 Conclusions

This study addressed the challenges posed by the prevalence of asymptomatic individuals during the COVID-19 pandemic, which undermined the reliability of epidemiological statistics in policymaking. While our earlier works of *contagion potential* (CP) as a continuous metric to quantify infection risk within a geographical region represented a significant advancement, CP estimation is hindered by incomplete or biased incidence data due to underreporting and testing constraints, making direct estimation infeasible. We employed a hypothesis-testing approach that infers CP from sampled data and also introduced an adjustment factor to calibrate the sample CP inferred from partially observed spatial contact data for an accurate estimation of population CP. Furthermore, we corrected the biases in epidemiological and mobility data, arising from heterogeneous reporting rates and sampling inconsistencies, through *inverse probability weighting*. By leveraging a spatial model for infection spread through social mixing and an optimization framework based on the SIRS epidemic model, we established the feasibility of estimating CP with high confidence using real infection datasets from Italy, Germany, and Austria. Our findings highlight how statistical methods can effectively correct for bias, social mixing, and sampling inconsistencies, ultimately strengthening CP as a reliable tool for outbreak mitigation strategies despite uncertainties and biases in epidemiological data.
